# Hyponatremia: An Untimely Finding in Ovarian Hyper-Stimulation Syndrome

**DOI:** 10.7759/cureus.16556

**Published:** 2021-07-22

**Authors:** Mohammed Elmahal, Petras Lohana, Priyanka Anvekar, Manoj K Menda, Arti .

**Affiliations:** 1 Endocrinology and Diabetes, London University, London, GBR; 2 Internal Medicine, Liaquat University of Medical and Health Sciences Hospital, Karachi, PAK; 3 Internal Medicine and Surgery, Mahatma Gandhi Mission (MGM) Medical College and Hospital, Mumbai, IND; 4 Medicine, St Johns National Academy of Health Sciences, Bangalore, IND; 5 Internal Medicine, Liaquat University of Medical and Health Sciences, Kunri, PAK

**Keywords:** hyponatremia, ohss, polycystic ovary syndrome (pcos), ascites, in vitro fertilization (ivf)

## Abstract

In-vitro fertilization (IVF) is one of several methods to assist individuals to conceive. This is a case of a young lady who presented to the emergency department with a one-day history of mild abdominal pain, vomiting, and tiredness. She had undergone uneventful IVF with an embryo transfer procedure in a specialized center three days earlier. The initial clinical examination was unremarkable, but laboratory investigations showed low serum sodium and albumin levels with relatively high leucocyte count. Considering the recent history of IVF coupled with the deranged laboratory results, the patient was admitted to the hospital for further assessment and evaluation. On the second day, the patient developed severe abdominal pain and distension and she became more distressed. A diagnosis of ovarian hyperstimulation syndrome (OHSS) was established and the patient was admitted to the intensive care unit (ICU) as her condition deteriorated thereafter. Twelve days later, the patient was discharged from the hospital after a full recovery.

## Introduction

As the number of people undergoing IVF is increasing, the complications of this procedure are expected to rise [1]. Ovarian hyperstimulation syndrome (OHSS) should always be anticipated in patients who have had recent in-vitro fertilization (IVF) and some of the alarming signs in life-threatening situations include enormously enlarged ovaries, a large volume of ascites, electrolyte disturbances and decreased blood pressure and urine output [2]. The interesting point of this case was the early derangement of the laboratory results particularly hyponatremia. This case shows that a high clinical index of suspicion is needed to early diagnose this disorder and consequently reduce the morbidity and mortality rates. Moreover, it demonstrates that hyponatremia can be an early laboratory finding to be encountered in OHSS.

## Case presentation

A 34-year-old lady with a history of failed IVF procedure one year back presented to the emergency department with one day mild epigastric pain, vomiting, and fatigue. Three days earlier, the patient had IVF with an embryo transfer procedure in a specialized center. She was well after the procedure; thus discharged home. She had normal menarche at the age of 14 years with regular menses, but she complained of oligomenorrhea for the preceding six years. She had been on metformin tablets 500 mg polycystic ovary syndrome for more than three years and was recently started on progesterone 10 mg to prepare the endometrium for conception. On examination, she looked exhausted but not pale or jaundiced. She was apyrexial, with a blood pressure of 128/70 mmHg, pulse rate 108 per minute (regular), and respiratory rate of 20 per minute. There was mild tenderness over the epigastrium but without signs of abdominal guarding and had normal bowel movements. On the second day, the intensity of the abdominal pain increased and the abdominal girth, and the patient became more tachypneic (respiratory rate 26 per minute). Nevertheless, the patient maintained adequate oxygen saturation throughout her hospital stay and did not require assisted ventilation.

The following table shows the investigations done on the first day of admission (Table 1).

**Table 1:** Initial investigation on the first day of admission HCG - human chorionic gonadotropin

Investigation	Result	Normal reference value
Total leucocyte count (×103/mL)	13.50	4.0-11.0
Hemoglobin (gm/dL)	12.3	12.0-15.0
Hematocrit %	43	36-44
Platelet (×103/mL)	372	150-450
C-reactive protein (mg/L)	3.8	0.0-7.5
Sodium (mmol/L)	127	135-145
Potassium (mmol/L)	4.6	3.6-5.10
Chloride (mmol/L)	97	98-107
Carbon dioxide (mmHg)	18.3	23.0-29.0
Bicarbonate (mmol/L)	28.5	22.0-32.0
Random glucose (mmol/L)	6.8	4.0-7.8
Urea (mmol/L)	2.6	2.0-7.1
Creatinine (µmol/L)	72	53-97
Gamma-glutamyltransferase (IU/L)	22	0-30
Total protein (gm/L)	53	61-79
Albumin (gm/L)	25	35-52
Total bilirubin (µmol/L)	17	5-21
Alanine transferase (IU/L)	59	14-54
Alkaline phosphatase (IU/L)	74	42-98
Prothrombin time (seconds)	12.7	10-14
Partial thromboplastin time (seconds)	39	28-40
Beta HCG (mIU)	158.5	<5
Serum thyroid-stimulating hormone (mIU/L)	1.8	0.4-4
Serum osmolality (mOsm/kg)	289	275-295
Urine osmolality (mOsm/kg)	280	50-1,200
Serum cortisol lvel (mcg/dL)	15	6-23

**Figure 1:**
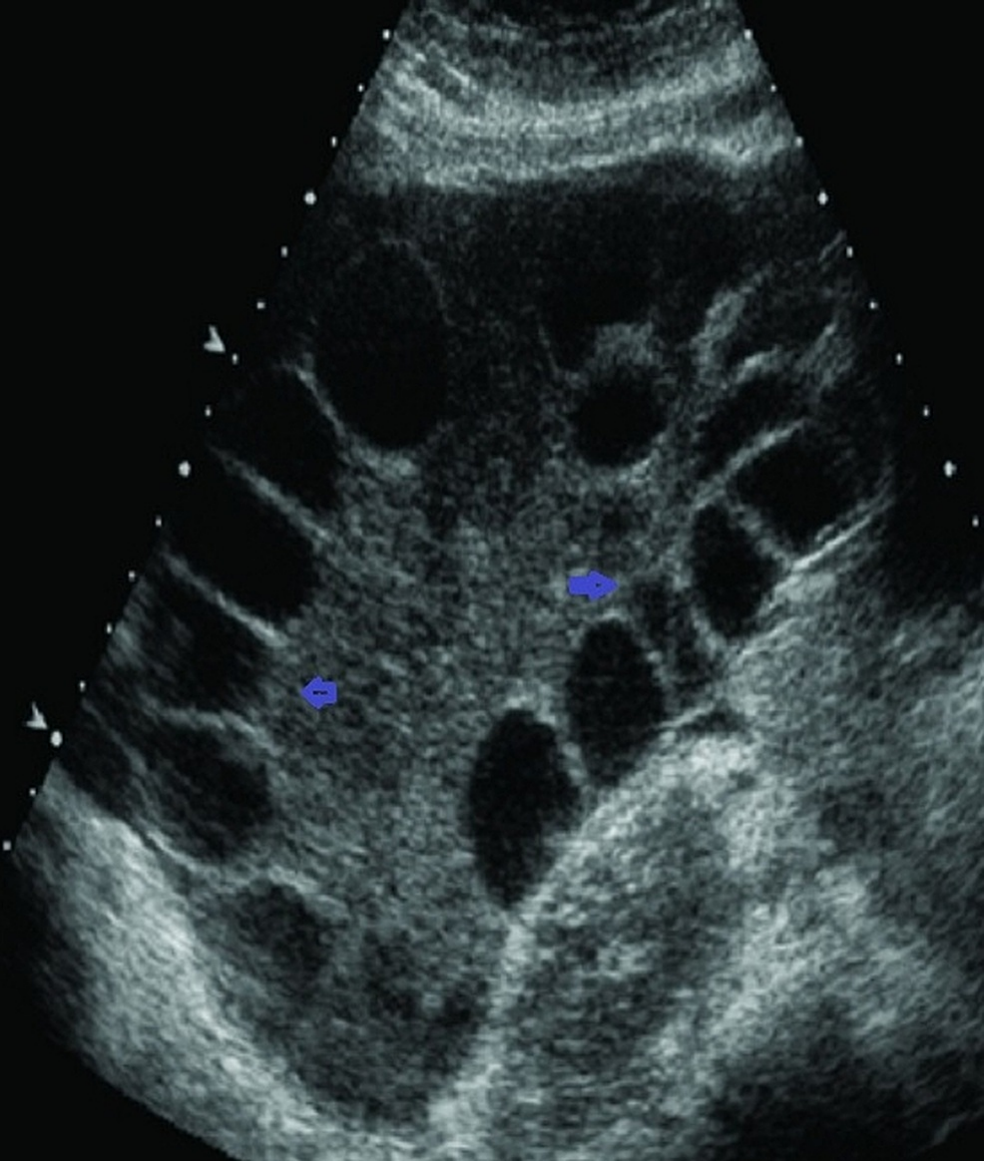
Multiple follicular cysts appreciated on the abdominal ultrasound

The relatively high white blood cell count in the presence of normal temperature and C-reactive protein (CRP) levels may be due to either hemoconcentration or stress response [3,4]. Urine analysis and culture were normal. Chest x-ray was within normal limits. Abdominal ultrasound showed multiple large cystic follicles in both ovaries with a moderate amount of free peritoneal fluid. Imaging of the visceral organs was unremarkable with no evidence of pleural effusion. Based on the recent IVF history and clinical picture, OHSS is the most likely diagnosis; and hyponatremia is a consequence of this disorder. The patient was initially treated conservatively with Isotonic saline, i.e., 0.9% saline. Ranitidine 50 mg and low molecular weight heparin 40 mg subcutaneous injection were given to guard against stress ulcers and thromboembolism. On the third day, the patient became more distressed with the rapid accumulation of ascites, so she was transferred to the ICU, where abdominal paracentesis of 2,000 mL of ascetic fluid was done. Therapeutic paracentesis with albumin replacement was done twice on the first five days of admission with total removal of 5,000 mL of the ascitic fluid. Dihydrogesterone 10 mg tablets BID continued to support the luteal phase to maintain the implanted embryo. After the eighth day of admission, the abdominal girth returned to normal measurement with no further rebound distension, and serial abdominal ultrasound showed shrinkage of the previously enlarged follicular cysts (Figure 1).

Fluid balance was assessed regularly, and the intravenous fluids were tapered gradually as the patient started oral intake. After 12 days of admission, the patient was discharged home. Recent abdominal ultrasound showed that the implanted embryo was viable.

## Discussion

OHSS is an iatrogenic complication of ovarian stimulation of assisted reproductive technology, e.g., IVF. It is marked by cystic enlargement of the ovaries due to exogenous gonadotropin stimulation and brisk fluid movement from the intravascular space to the third space, particularly the abdominal cavity [1,5]. The increased capillary permeability is the key pathophysiologic feature which is basically due to human chorionic gonadotropin (hCG) [1,5,6]. Additionally, cytokines and vasoactive peptides, such as prostaglandins, the renin-angiotensin-aldosterone system, insulin-like growth factor-1, interleukin-6, and inhibin, have been involved in this process [1,6]. The vasoactive peptide vascular endothelial growth factor (VEGF) is considered the main mediator in vascular permeability. It seems that hCG induces VEGF secretion in a dose and time-dependent pattern as further doses of hCG do not affect the upregulation of VEGF [7]. It has been shown that VEGF and its receptor VEGF-2 are elevated considerably in response to hCG, and the greatest vascular permeability is directly related to the levels of VEGF [1,5-8]. After its secretion from the ovarian granulose-lutein cells, VEGF repositions the actin endothelial fibers, particularly cadherin and claudin-5, and eventually increases vascular permeability [1,9]. Factors, such as young age, previous OHSS history, reduced body weight, polycystic ovaries, exposure to high doses of gonadotropins, and high serum estradiol levels, may increase the likelihood of developing OHSS [1,5,6].

Diagnosis of OHSS requires a recent history of ovarian stimulation, followed by either ovulation or exogenous hCG administration [6]. OHSS can be graded into mild, moderate, severe, and critical forms based on the clinical presentations, laboratory and imaging findings [6]. It can also be classified into early or late OHSS depending on the time of onset, and this classification is particularly of prognostic value. Early OHSS occurs within 10 days of hCG administration and shows an exaggerated response to gonadotropin stimulation. It is a rare complication, being usually less severe than the late OHSS. On the other hand, late OHSS occurs after 10 days of hCG administration; it resembles a continuous effect of endogenous hCG stimulation from the time of early gestation [1,4].

It was found that most patients with OHSS show high hematocrit value, reflecting the hemoconcentration status, which is secondary to hypovolemia; it is more reliable than high white blood cell count in assessing the severity of OHSS [1].

Hyponatremia, hypoalbuminemia, and other electrolyte disturbances reflect the leakage consequences of the intravascular fluid with its contents into the third compartments, particularly the peritoneal space [3]. Hyponatremia is seen in more than 50% of patients with OHSS and is thought to be due to antidiuretic hormone (ADH) hypersecretion. The latter occurs secondary to decreased intravascular volume to maintain adequate arterial blood volume. In our patient, we ruled out the syndrome of inappropriate ADH (SIADH) on the basis of laboratory investigations. Furthermore, as OHSS represents a hypovolemic state, secondary hyperaldosteronism ensues with subsequent activation of the renin-angiotensin-aldosterone system [1,10].

Deranged liver function is commonly observed in a patient with OHSS. The exact pathophysiologic mechanism underlying these changes is unknown, but two hypotheses may explain the elevation of aminotransferases in one-third of patients with OHSS. First, the histological changes seen in the hepatocytes are similar to those seen in pregnancy, which may point to the role of estradiol as a causative agent of this disorder. Second, increased vascular permeability may lead to hepatic edema with subsequently elevated liver enzymes. Besides, the circulatory dysfunction may further enhance liver tissue ischemia and eventually defective liver function. Nevertheless, the liver function returns to normal after the resolution of OHSS in most patients [4].

It is a challenging task to predict OHSS in individuals depending only on their risk factors. Serum levels of anti-Müllerian hormone (AMH) are directly correlated with the ovarian response throughout IVF cycles; an observation can be utilized in predicting OHSS by checking basal AMH before hCG administration [11]. It has been demonstrated that mutations of follicular-stimulating hormone (FSH) receptor genes play a role in OHSS; however, their role in predicting OHSS needs to be explored [1].

Treatment of OHSS depends on the severity of symptoms and clinical and laboratory findings (e.g., hypotension, hemoconcentration, electrolyte, and liver function disturbance). Since this disorder is self-limiting, conservative treatment is the general approach. If pregnancy is not contemplated, OHSS takes around two weeks to resolve as hCG decreases by this time [3]. Mild and moderate OHSS can be treated on an outpatient basis with simple analgesics and careful assessment and monitoring to guard against any progression to the severe form. When hospitalization is warranted, thorough reassessment is essential. However, a manual pelvic examination should be avoided because of the risk of ovarian rupture [5].

Treatment of hypovolemia, hypotension, and electrolyte disturbance is a priority. OHSS is associated with reduced mean arterial pressure, which necessitates rapid correction with intravenous fluid. However, fluid therapy may aggravate edema and ascites. Therefore, intravenous fluids should be given with great caution and volumes sufficient to keep urine output around 30 mL/hr. Given that these patients are commonly hypovolemic and hyponatremic, normal saline and/or five percent dextrose in normal saline are the favorite intravenous fluids [3,5]. Albumin, which is usually used later in treatment, is the favorite plasma expander, particularly when there is hypoalbuminemia [5]. Moreover, it was found to decrease the severity of OHSS if given in the early stages of the disease [3].

Individualizing the treatment of controlled ovarian stimulation is the safest approach to reduce the possibility of OHSS. For instance, starting with the selective estrogen receptor modulator (SERM), i.e., clomiphene citrate with or without FSH, can be used initially in selected women [1]. Withholding hCG is the most effective preventative measure against OHSS. This approach is usually applied to women with several risk factors; however, this method prevents ovulation [1].

Several studies have suggested that OHSS can be prevented by using gonadotropin-releasing hormone (GnRH) agonists instead of hCG for maintaining oocyte maturation. It has been shown that using this method can completely prevent OHSS, but this is done at the expense of pregnancy outcomes and birth rates [1]. A review of several randomized clinical trials proposed that GnRH agonists should not be regularly used to activate oocyte maturation but can be used in selected women with a high risk of OHSS [9].

Coasting is a policy of delaying hCG administration in women who show high estradiol levels in response to ovarian stimulation. Therefore, hCG is given later when estradiol reaches a relatively safe level [8]. Nevertheless, the emergence of the new long-acting follicular stimulants has made coasting less preferred, as they replaced the daily injections of gonadotropin throughout the first week of controlled ovarian stimulation. Most importantly, they significantly reduced the incidence of OHSS. Moreover, there is no sufficient evidence to support coasting as a preventative measure [1]. Another measure is the use of only progesterone instead of progesterone and hCG to support the luteal phase. This method has markedly decreased the incidence of OHSS [1]. Low-dose aspirin started on the first day of ovarian stimulation has shown a significant reduction in OHSS. This effect may be attributed to the effect of aspirin in reducing the expression of VEGF [8]. Although the renin-angiotensin system is involved in the pathogenesis of OHSS, drugs that block this system may result in a deleterious effect on both mother and fetus [12].

There is evidence supporting the use of dopamine agonists (e.g., cabergoline) on the day of hCG administration to decrease the incidence of OHSS. These drugs suppress the phosphorylation of VEGF-2 receptors, which occurs in response to hCG administration [1].

OHSS is associated with disrupted osmoregulation, hypovolemia, and electrolyte imbalance; these changes occur much earlier than previously thought [3]. Hyponatremia could be considered a red herring that may herald the full clinical picture of OHSS.

## Conclusions

Hyponatremia is a common early laboratory finding in OHSS. Symptoms of OHSS are frequently non-specific. So, clinicians should have a high clinical index of suspicion to early diagnose and treat this disorder. Cytokines and vasoactive peptides (e.g., VEGF) play a major role in the pathogenesis of OHSS. Prevention of OHSS can be achieved by careful assessment of risk factors and therefore individualized ovarian stimulation strategy.
